# An automated image analysis method to measure regularity in biological patterns: a case study in a Drosophila neurodegenerative model

**DOI:** 10.1186/s13024-015-0005-z

**Published:** 2015-03-12

**Authors:** Sergio Diez-Hermano, Jorge Valero, Cristina Rueda, Maria D Ganfornina, Diego Sanchez

**Affiliations:** Instituto de Biología y Genética Molecular-Departamento de Bioquímica y Biología Molecular y Fisiología, Universidad de Valladolid-CSIC, c/ Sanz y Forés 3, Valladolid, Spain; Center for Neuroscience and Cell Biology. Mitochondrial Dysfunction and Signaling in Neurodegeneration group. University of Coimbra, Coimbra, Portugal; Instituto de Matemáticas (IMUVA), Universidad de Valladolid, Valladolid, Spain

**Keywords:** Eye classifier, ImageJ plugin, Neurodegeneration, Ommatidia

## Abstract

The fruitfly compound eye has been broadly used as a model for neurodegenerative diseases. Classical quantitative techniques to estimate the degeneration level of an eye under certain experimental conditions rely either on time consuming histological techniques to measure retinal thickness, or pseudopupil visualization and manual counting. Alternatively, visual examination of the eye surface appearance gives only a qualitative approximation provided the observer is well-trained. Therefore, there is a need for a simplified and standardized analysis of fruitfly eye degeneration extent for both routine laboratory use and for automated high-throughput analysis. We have designed the freely available ImageJ plugin FLEYE, a novel and user-friendly method for quantitative unbiased evaluation of neurodegeneration levels based on the acquisition of fly eye surface pictures. The incorporation of automated image analysis tools and a classification algorithm sustained on a built-in statistical model allow the user to quickly analyze large sample size data with reliability and robustness. Pharmacological screenings or genetic studies using the Drosophila retina as a model system may benefit from our method, because it can be easily implemented in a fully automated environment. In addition, FLEYE can be trained to optimize the image detection capabilities, resulting in a versatile approach to evaluate the pattern regularity of other biological or non-biological samples and their experimental or pathological disruption.

**Electronic supplementary material:**

The online version of this article (doi:10.1186/s13024-015-0005-z) contains supplementary material, which is available to authorized users.

## Introduction

The retinal system in Drosophila has been for long a very useful model to study the pathogenic mechanisms of human neurodegenerative diseases [1] and to test the efficacy of phenotypic modifiers of a pathological condition that arise from diverse genetic screenings [2]. The fruitfly compound eye is formed by around 800 units, called ommatidia, which display a very regular pattern. The stereotypic development of the insect retina and the extensive knowledge of the cellular and molecular mechanisms involved, make this a very reliable system to evaluate whether altering the expression of mutated proteins is linked to cell degeneration.

Poly-glutamine-based neurodegenerative diseases, such as Huntington’s and a number of Spinocerebellar ataxias, have been studied using the Drosophila retina as a test tube and the Drosophila GAL4-UAS system for transgene expression [3-5]. A qualitative examination of the external appearance of the fly eye (described as rough-eye phenotype) has been widely used to categorize whether a mutation would improve or worsen a given degeneration level. However, most quantitative estimates have relied upon methods involving tissue fixation, paraffin or cryostat sections, histochemical/immunohistochemical staining or scanning/transmission electron microscopy (SEM/TEM). Using these preparations, researchers have evaluated retinal thickness, rhabdomere counting, the regularity of the hexagonal photoreceptors array, or have scored for the presence of expected features in the retinal surface [6-10].

In this work we present a simple and reliable method to quantify the degree of retinal degeneration based on fly eye surface photographs. An earlier version of this method was successfully used to assess the rescuing ability of Lipocalin genes and its dependence on autophagic activity in a model of Type I Spinocerebellar Ataxia [11]. Following tests with other commonly used techniques, and validation with randomly chosen eye pictures, our image analysis method was implemented in a freely distributed plugin (FLEYE) for the open source image analysis program Fiji [12]. Our user-friendly Fiji plugin allows for a fast evaluation of the eye regularity pattern, and for a quantitative unbiased assessment of neurodegeneration under diverse pathogenic levels or genetic penetrance conditions. Since the method obtains a regularity index for each image, it could also be applied to study changes in any regular biological pattern.

## Methods

### Fly lines and maintenance

Flies were grown in a temperature-controlled incubator at 25°C, 60% relative humidity, under a 12 h light–dark cycle. They were fed on wet yeast 84 g/l, NaCl 3.3 g/l, agar 10 g/l, wheat flour 42 g/l, apple juice 167 ml/l, and propionic acid 5 ml/l. Fly females were used in all experiments. We used the line gmr:GAL4 to drive transgenes expression to the eye photoreceptors. UAS:hATXN1^82Q^ was used to trigger the neurodegenerative phenotype [13] and different UAS:modifier-gene constructs were used to test the system.

### Histological methods

Adult fly heads were fixed with 4% paraformaldehyde, dehydrated in ethanol, and included in paraffin. Paraffin sections (4 μm) were dewaxed with xylene and rehydrated in an ethanol series. Histochemical staining with hematoxylin and eosin was performed according to standard procedures. The labeled sections were photographed with an Eclipse 90i (Nikon) fluorescence microscope equipped with DS-Ri1 (Nikon) digital camera. Images were acquired with the NIS-Elements BR 3.0 software (Nikon), and processed with ImageJ (version 1.48p).

### External eye surface digital imaging

Digital pictures (1280x960 pixels) of the surface of fly eyes were taken with a DS-L1 digital camera, in a Nikon SMZ1000 stereomicroscope equipped with a Plan Apo 1x WD70 objective. The flies were anaesthetized with CO_2_ and frozen for 10 minutes at −20°C. Their bodies were immobilized on dual adhesive tape, and their heads set up to have an eye parallel to the stereomicroscope objective. Fly eyes were illuminated with a homogeneous fiber optic light (20 W; KL 200, Zeiss). A white balance was performed on the background white surface. Additional settings include a 6x optical zoom in the stereomicroscope that results in a final resolution of 1.85 μm/pixel. Image files were saved in Tiff format.

Special care must be put into maintaining the same illuminating conditions and camera settings between experiments, as differences among pictures of the same stack may introduce undesired artifacts that would hamper the discrimination capacity of FLEYE.

### Statistical analysis

The statistical analyses and graphical outputs for the measurements described below were performed and generated using SAS 9.2 and SPSS.

Multiple Box plots and histograms, and dispersion diagrams and pairwise sample correlations have been used to describe variable distributions within groups and pairwise relationship respectively.

Differences between eyes with different degeneration degrees were assayed using ANOVA for the selected variables describing the degree of regularity in each image (see below). A principal component analysis (PCA) was performed and the scores of the first principal component were used to generate clusters. A Multinomial Logistic Model for the clusters defined previously, was fitted using only a subset of the data set (considered as the training data). The remaining data were used as a test to validate the model. The final set of predictive variables was selected using a stepwise variable selection approach, and the estimated probabilities of each image to belong to each cluster were used to derive a regularity index (IREG) adopting values from 0 to 1. The robustness of the procedure was tested by randomly splitting the data set in different ways (in training and test sets) and comparing the resulting regularity indexes.

Final comparisons of IREG medians between different experimental classes (genotypes) were performed using Kruskal-Wallis non-parametric hypothesis contrast. Post-hoc tests followed the Dunn’s method. A p-value <0.05 was considered statistically significant.

### FLEYE plugin requirements

The FLEYE plugin is composed of four different ImageJ1 macros: FLEYE_menu_v2.ijm, Fleye_ROISv1.2.ijm, Fleye_optimizer_v4.2.ijm and Fleye_v10.2.ijm. It has been developed in the ImageJ version 1.49d, using Fiji. The plugin uses the “Bio-Formats Importer”, released by the OME Consortium (http://openmicroscopy.org) to be able to open the different formats of image files. The ‘Bio-formats Importer’ plugin is included in the Fiji package. Thus, we recommend using this package to run FLEYE.

The FLEYE plugin pack, a quick guide and user manual, and the GNU general public license file are provided in Additional files 1, 2, 3, and can be accessed at http://imagejdocu.tudor.lu/doku.php?%20id=plugin:analysis:fleye:start&#fleye.

## Results and discussion

### Retinal degeneration in a Drosophila model of human spinocerebellar ataxia SCA1

Poly-glutaminated proteins are a common pathogenic mechanism of a diverse array of human neurological diseases. Here we have used the GAL4/UAS system to express a pathogenic version of human Ataxin 1 with an expanded glutamine tract (hATXN1^82Q^) in Drosophila retinal photoreceptors using the gmr:GAL4 driver. In this model of SCA1, photoreceptors accumulate nuclear inclusions of the human protein and start degenerating during late pupal stage when flies develop at 25°C [13]. After expressing the mutated pathogenic protein form, the adult retina degenerates and the eye surface morphology loses its regular pattern appearance [13].

**Figure 1:**
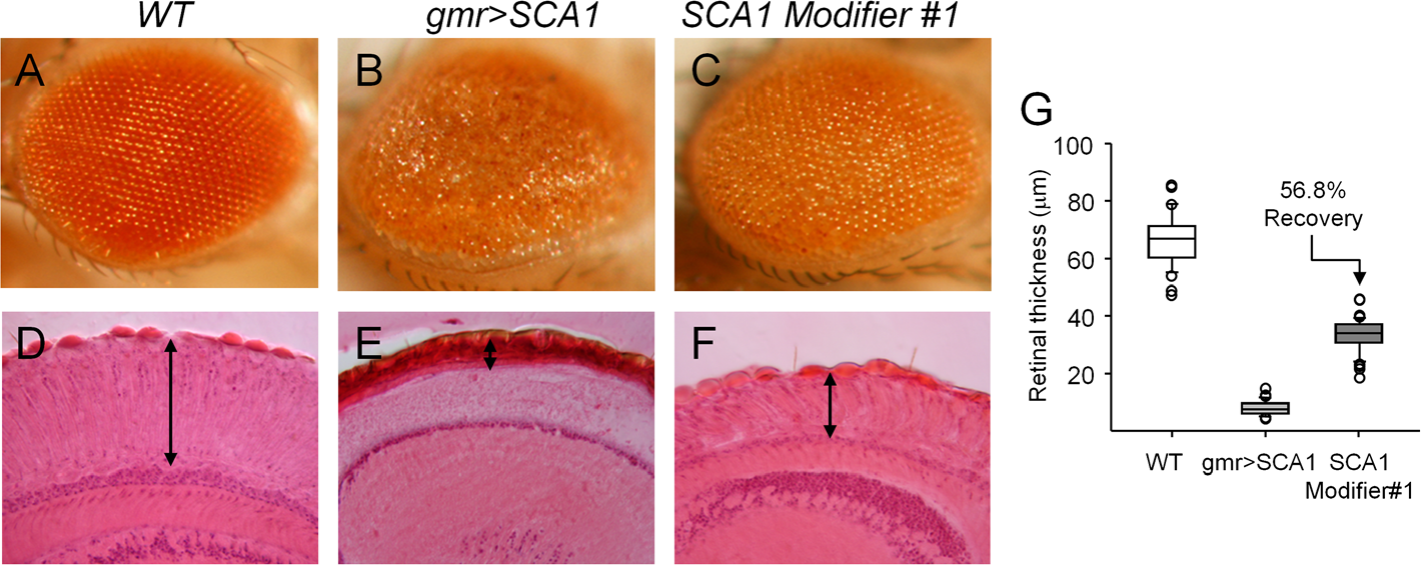
**Histological method to analyze Drosophila eye degeneration phenotypes.** External pictures of fly eyes surface **(A-C)** and histological measurement of retinal thickness **(D-F)**. Images in a column correspond to the same genotype. **(G)** Retinal thickness quantification in μm, resulting in a significant recovery from the degenerated genotype for the *gmr > SCA1* Modifier#1 (n = 34-46 sections/genotype).

A qualitative assessment does indeed suffice to categorize a retina as healthy vs. degenerate (Figure 1A-B). However, to analyze the effect of genetic modifiers of the rough-eye phenotype (Figure 1C) or to estimate the outcome of a drug that could enhance/diminish the extent of degeneration, we must quantify the degree of the modification achieved. A standard way to obtain this estimate has been to measure retinal thickness after histological processing of fly retinas, which involves tissue fixation and sectioning followed by either immunolabeling with retinal markers or standard histochemical staining (Figure 1D-G). Aside of the complex and time consuming methodological procedures, the sectioning angle and the variable thickness of different regions of the degenerated retina introduce a high degree of variability in the measurements.

### Image processing

We have designed a digital image processing to automatically detect the bright spots that appear in the image due to light reflection in ommatidia.

**Figure 2:**
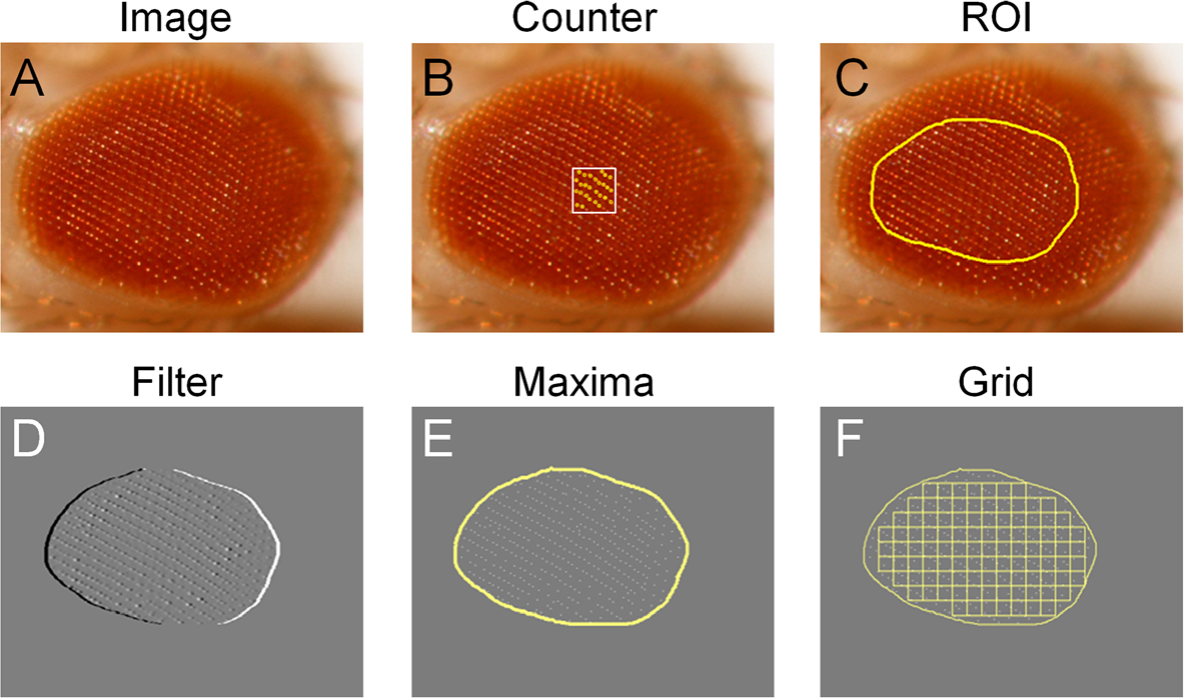
**Representation of image processing steps performed by FLEYE plugin.** Starting with eye surface images **(A)** a “training step” is performed where the plugin detection is fitted to the number of user-counted ommatidia in WT eyes **(B)**. Then, the user defines a region of interest (ROI) **(C)**. An averaging step (Filter) normalizes the picture **(D)** and pixel maxima are identified **(E)**. A squared grid is applied to the single-pixel maxima image **(F)**. The final variables are obtained either globally from the distance between maxima or locally from spatial information of the pixel distribution in every cell of the grid.

In a first step, the image is converted to 8-bit grayscale (Figure 2A). The user must then define a region of interest (ROI) delimiting the area of the eye that is in focus for each image (Figure 2C). Only the ROI will be subjected to subsequent analysis. A subtracting-background step is performed using the Fiji plugin “Subtract Background” and a surface-like filter applied to increase the contrast of bright spots. The surface-like filter duplicates the image, inverts the duplicate, displaces it horizontally and then creates a new image by averaging the pixel intensity values of the original and duplicated images (Figure 2D). This procedure normalizes the images by removing any disturbances introduced by variations in the intensity values of the original colored images.

The next step involves finding the maxima of the intensity pixel function with the “Find maxima” Fiji plugin using a predetermined tolerance value. In our case, these maxima approximate the position of fly ommatidia (Figure 2E). Therefore, counting the number of maxima gives an estimation of the total number of ommatidia present in the ROI selected area.

An optional optimization step is also available. This step requires the user to manually identify ommatidia in representative areas of eye images (Figure 2B) to calibrate two main detection parameters: the tolerance of the “Find maxima” plugin and the rolling ball radius of the “Subtract Background” plugin.

### Maxima analysis

Our image analysis aims at detecting discrepancies in the regularity of the spatial distribution of ommatidia. Our strategy follows two main processes: a global one, based on distances between maxima, and a local one, that splits the ROI into a grid of squared cells and extracts statistical and spatial information of the maxima in each grid-cell.

Once the coordinates of the local maxima are obtained, we calculated the distance of each single maxima to its “nearest neighbor” (in normalized units to fit with our statistical model) using a self-developed algorithm.

Finally, to extract local spatial information, the squared grid (with a cell size set either by default or by the user) is applied to the single-pixel maxima image. In each different grid-cell we calculated the number of maxima per cell and different spatial parameters that are influenced by the distribution of maxima within each cell (distance between center of the cell and maxima center of mass, skewness and kurtosis). Note that the image regions near the edge of the original user-defined ROI are discarded, as FLEYE only takes into account the squares that fit completely into the ROI (Figure 2F). All the variables (global or grid-cell-based, Table 1) were used to develop a statistical model.

**Table 1:** **List of the 18 variables used for the statistical model development**

	**Variables**			
*Grid-based variables:*				
Grid-Cells %	N_maxima_<2	N_maxima_<4	N_maxima_<6	N_maxima_<8
Maxima per cell	Mean†	Variance†	Skewness†	Total†
Distance to the center of mass	Mean†	Variance†	Skewness†	
Kurtosis	Mean	Variance	Skewness	
Skewness	Mean	Variance†	Skewness	
*ROI-based variables:*				
Nearest Neighbor		Variance†		

Each row accounts for a given spatial measurement of the point distribution per grid cell or per ROI. *Grid*-*Cells (%)* refers to the percentage of cells with less than a certain number of maxima (2, 4, 6 or 8). Variables marked with † passed the correlation test and were tested in the ANOVA analysis (Table 2).

### Statistical methods

To estimate the degree of retinal degeneration we followed a statistical procedure based on two steps: 1) Categorization of the degeneration extent in discrete classes and estimation of the probability of an eye to belong to a certain class; 2) Computation of the overall regularity degree of each eye as a weighted mean of those probabilities.

Our image sample includes 152 fly eyes of five different experimental groups, with a retina surface displaying a morphology gradient from strong degeneration to a wild type state. The fly genotypes were not introduced in the modeling steps, which were blind to the assignment of each image to the model-generated clusters. As described above, we first extracted 18 mathematical variables from the processed images (Table 1). A correlation analysis reduced the number to 9 variables, eliminating those with very high and significant correlation indexes with at least one of the remaining variables. Differences between eyes with different degeneration degrees were then tested using ANOVA for the 9 variables selected (Table 2).

**Table 2:** **Test of the discrimination power of 9 selected variables**

		**Sum of squares**	**Degrees of freedom**	**Mean squares**	**F**	**Significance**
TOTMAX	Between-groups	313102.269	2	156551.135	37.026	0.000
	Within-groups	629991.204	149	4228.129		
	Total	943093.474	151			
MPCM	Between-groups	13.228	2	6.614	52.478	0.000
	Within-groups	18.779	149	0.126		
	Total	32.007	151			
MPCVAR	Between-groups	0.99	2	0.495	7.345	0.001
	Within-groups	10.041	149	0.067		
	Total	11.031	151			
MPCSKEW	Between-groups	6.51	2	3.255	33.911	0.000
	Within-groups	14.302	149	0.096		
	Total	20.812	151			
SKEWVAR	Between-groups	66.349	2	33.175	9.356	0.000
	Within-groups	528.341	149	3.546		
	Total	594.69	151			
DISTM	Between-groups	37.854	2	18.927		
	Within-groups	62.047	149	0.416	45.451	0.000
	Total	99.901	151			
DISTVAR	Between-groups	79.766	2	39.883		
	Within-groups	386.099	149	2.591	15.391	0.000
	Total	465.865	151			
DISTSKEW	Between-groups	5.542	2	2.771	37.751	0.000
	Within-groups	10.937	149	0.073		
	Total	16.479	151			
LOGNNVAR	Between-groups	17.848	2	8.924	49.302	0.000
	Within-groups	26.97	149	0.181		
	Total	44.817	151			

ANOVA test was performed on 9 out of 18 variables that passed the correlation test and resulted in reasonable discrimination between degeneration groups by PCA analysis. *TOTMAX* is the total number of maxima detected per image. *MPCM, MPCVAR* and *MPCSKEW* refer to the mean, variance and skewness of the maxima per cell, respectively. *SKEWVAR* is the skewness of the intensity values variance per cell. *DISTM, DISTVAR* and *DISTSKEW* refer to the mean, variance and skewness of the centroid-to-mass-center distance, respectively. *LOGNNVAR* is the logarithm of the nearest neighbor variance. The between-groups and within-groups components of the variance are estimated computing the squared errors (sum of squares) and averaging by the degrees of freedom (df, obtained as *k-1* between groups, *N-k* within groups and *N-1* overall; where *k* is the number of groups involved, and *N* the sample size), thus resulting in the quadratic mean (${\widehat{s}}_b^2$ between groups and ${\widehat{s}}_w^2$ within groups). The F-value is ${\widehat{s}}_b^2/{\widehat{s}}_w^2$, whose significance is evaluated following a *F*_2,149_ distribution.

**Figure 3:**
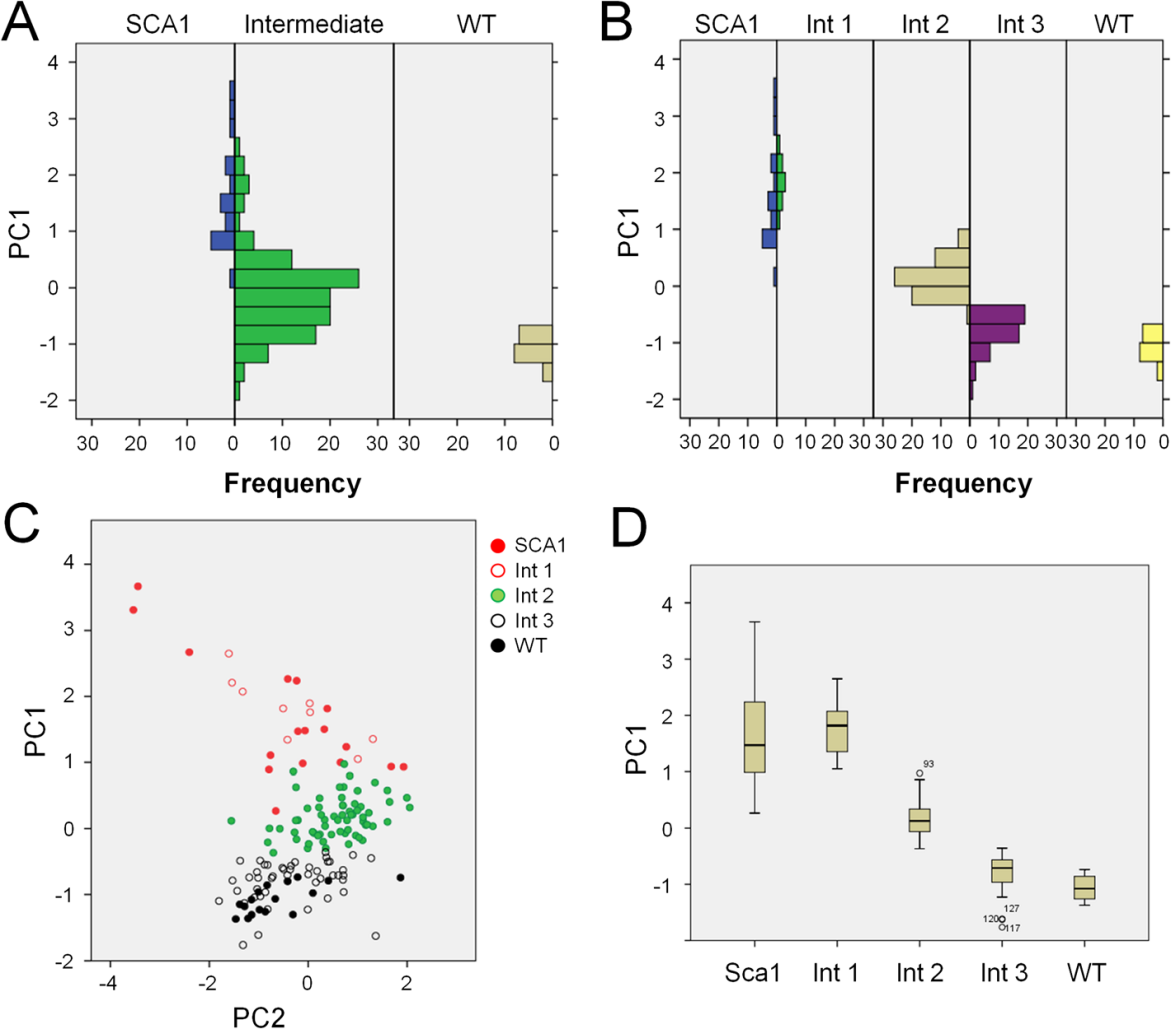
**Principal component analysis used to generate FLEYE built-in statistical model. (A)** The first component of a PCA analysis performed with 9 variables discriminates between WT and SCA1 degenerated eyes and shows a polymodal distribution of intermediates. **(B)** The first PCA factor splits the sample into five different categories, 0 to 4, ranging from a healthy eye (class 0) to a totally degenerated eye (class 4). The classification remains the same even after removing 4 redundant variables among the 9 used (not shown). **(C)** The first PCA component explains 60% of the total variance and constitutes a clearly discriminating tool **(D)**, whilst the second component explains 20% of the variance, but does not differentiate between healthy and degenerated samples.

A principal component analysis (PCA) was performed using the 9 selected variables (Figure 3). The first component (PC1) explains 60% of the variance, and the second component (PC2) 20% of the variance. PC1 clearly discriminates the wild type and fully degenerated eyes, but the distribution of PC1 scores in the intermediate group exhibits a multimodal pattern indicating a mixture of different groups within the intermediate level (Figure 3A). PC1 was used then to split the intermediate group into 3 different categories (Figure 3B-D). Five different categories are therefore considered in our data: Class 0 corresponds to wild type retinas; intermediate degeneration degrees belong to classes 1 to 3, and fully degenerated eyes are assigned to class 4.

**Figure 4:**
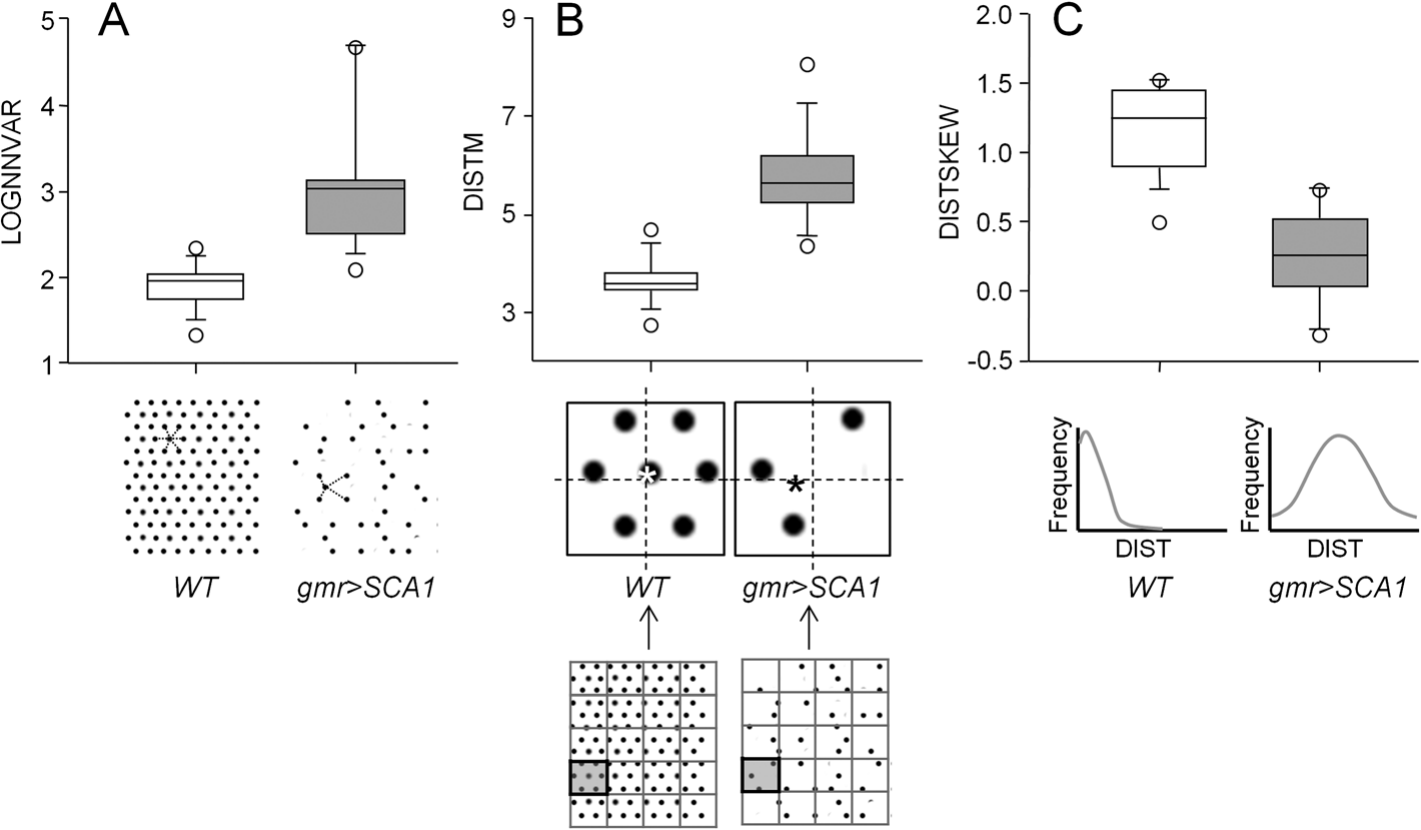
**Behavior of the three variables accounting for the predictive value of FLEYE statistical model.** Values of the three statistical model variables obtained in healthy (*WT*) and degenerated (*gmr > SCA1*) fly eye samples (n = 35/genotype). **(A)** LOGNNVAR variable operates over the whole ROI (grid-cell independent). The nearest neighbor distances are expected to be more similar to each other in a WT eye than in a degenerated one, which has lost regularity and hence distances display a higher variance. **(B)** The variable DISTM for a grid cell (a representative pair is enlarged and shown in gray in the grid) is calculated as the mean difference between the centroid (the center point of the cell, marked as the intersection of dashed lines) and the maxima center of mass (the brightness-weighted average of the x and y coordinates of all pixels in the image or selection, marked with asterisks). A WT eye is expected to have a more regular distribution of maxima in a cell than a degenerated one, thus resulting in a mass center value closer to the centroid (lower difference output). **(C)** The variable DISTSKEW for a grid cell represents an asymmetry measure of the distance between the centroid and mass center (third spatial moment). As low values of DIST are more frequent in WT, a right-tailed distribution is expected, resulting in a positive skew value.

To evaluate the probability for an eye to belong to a certain degeneration class, a Multinomial Logistic Regression was performed to estimate the parameters of a qualitative response model based on a categorical dependent variable “I” (degeneration class). The retinal samples were divided through random sampling into training and testing subsets, which were used to build the model and to validate it respectively. An automatic stepwise procedure reduced the number of variables to 3, which were found to be the most predictive factors for the model. Ultimately, the variables selected by this statistical procedure are: i) the logarithm of the variance of the “nearest neighbor” distance between maxima (LOGNNVAR) (Figure 4A), ii) the mean distance between the grid-cell centroid and maxima center of mass (DISTM) (Figure 4B), and iii) the skewness of DIST (DISTSKEW) (Figure 4C).

The model parameters are shown in Table 3. A total of 16 parameters are estimated and denoted by name_i, where name can be independent, distm, distskew, or lognnvar and i = 0,1,2,3.

**Table 3:** **Parameter values of FLEYE statistical model**

***Parameter i***	***independent***	***distm***	***distskew***	***lognnvar***
0	84.366	-13.681	20.391	-17.32
1	60.004	-11.097	15.005	-6.976
2	29.07	-4.448	7.663	-3.308
3	1.299	0.242	-17.746	-0.104
4	0	0	0	0

Values corresponding to each parameter in the multivariate logistic model (mathematical expression (1) in the text) for each PP_i_ (probability for a given retina to belong to a degeneration class, for i = 0,1,2,3,4).

Denoting PP_i_ as the probability for a given retina to belong to a certain class of degeneration (for i = 0,1,2,3,4), each PPi is defined in terms of the parameters as follows:

1 $$ \log \frac{P{P}_i}{P{P}_4}= indepen{d}_i+ dist{m}_i* DISTM+ dist ske{w}_i* DISTSKEW+ lognnva{r}_i* LOGNNVAR $$

For i = 0,1,2,3.

Therefore,

2 $$ \frac{P{P}_i}{P{P}_4}= \exp \left( indepen{d}_i+ dist{m}_i* DISTM+ dist ske{w}_i* DISTSKEW+ lognnva{r}_i* LOGNNVAR\right) $$

For i = 0,1,2,3.

Now, being *PP*_0_ + *PP*_1_ + *PP*_2_ + *PP*_3_ + *PP*_4_ = 1

We have that,

3 $$ P{P}_4=\frac{1}{{\displaystyle {\sum}_{i=0}^3} \exp \left({a}_i\right)+1}\  and\ P{P}_i=\frac{ \exp \left({a}_i\right)}{{\displaystyle {\sum}_{i=0}^3} \exp \left({a}_i\right)+1};\mathrm{i}=0,1,2,3. $$

Where,

$$ {a}_i= indepen{d}_i+ dist{m}_i* DISTM+ dist ske{w}_i* DISTSKEW+ lognnva{r}_i* LOGNNVAR $$

After training the model with a group of randomly selected retinas, we finally compute the overall retinal degeneration degree defining a regularity index, *IREG:*

4 $$ IREG = \frac{4*P{P}_0 + 3*P{P}_1 + 2*P{P}_2 + P{P}_3}{4} $$

where *IREG* = 1 accounts for total regularity (WT eye), whilst *IREG* = 0 means total absence of regularity (degenerated eye). As a result, the bigger the probability of belonging to a low degeneration class (PP_0_ and PP_1_) the closer the index moves to 1, and vice versa. Intermediate values ranging from 0 to 1 will represent partial degeneration cases or rescued genotypes.

**Figure 5:**
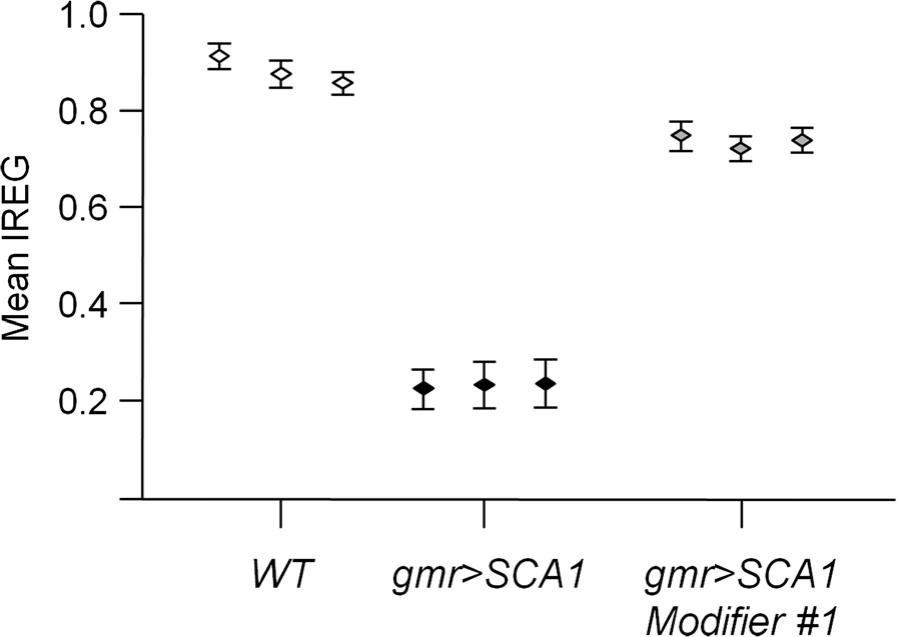
**IREG robustness test.** Mean IREG values obtained using three different training sets for WT, gmr > SCA1 and gmr > SCA1 Modifier#1. The robustness of the procedure is demonstrated due to the negligible changes in the computed values among trials.

To verify that the index obtained does not depend on the training set, three different divisions of training and test subsets were considered, and the corresponding IREG values obtained. The IREG distributions were very similar within the three groups: WT, SCA1 and modifiers (Figure 5), indicating the robustness of the procedure.

Although the large sample size used in our experiments is needed to develop and validate this method, FLEYE users should comply with standard experimental design guides to properly evaluate the sample size required in their experiments.

### Fiji macro design

**Figure 6:**
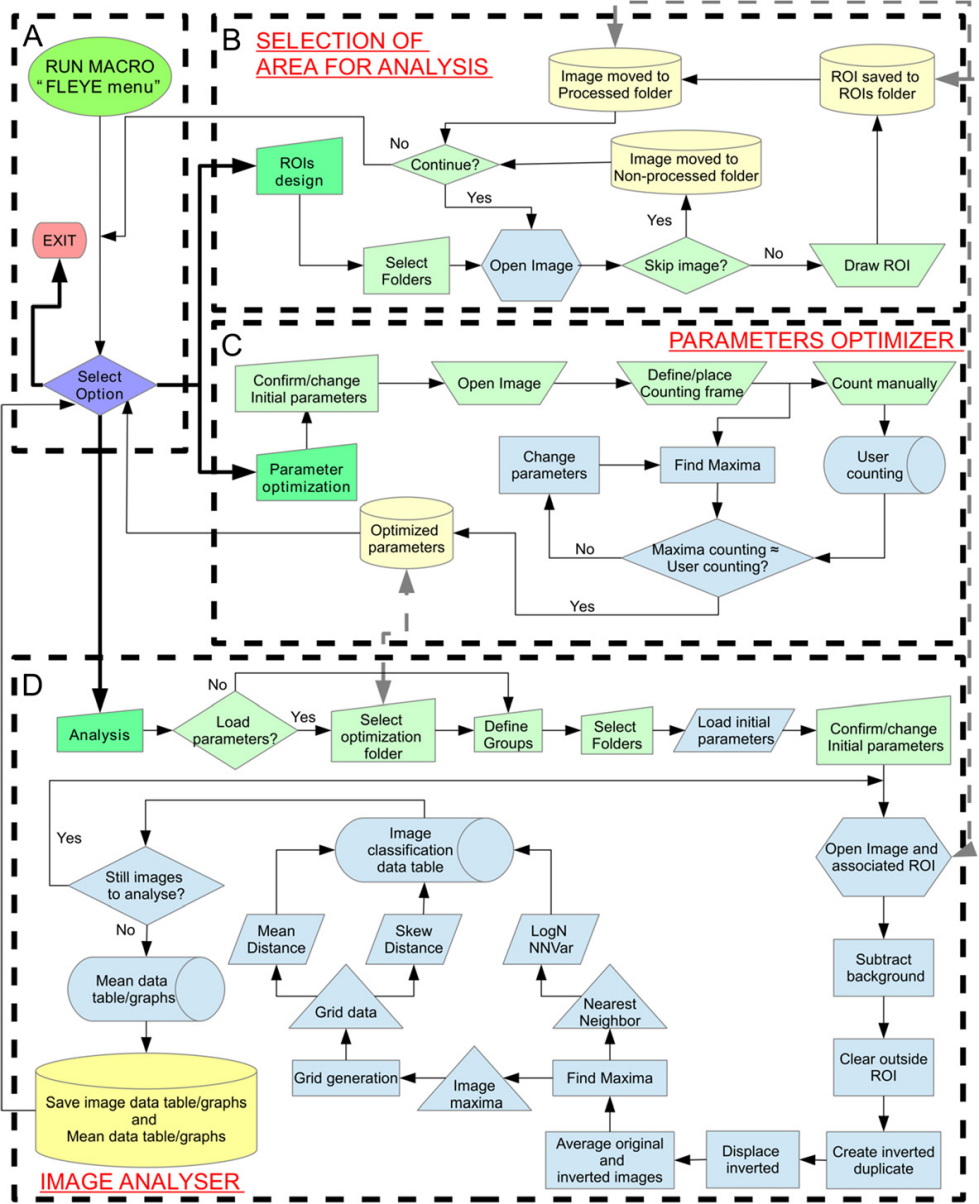
**FLEYE plugin algorithm flow chart.** Four different macros and the interaction between them are displayed. **(A)** Main hub to access any of the actual computing macros. **(B)** ROI processing and storage. **(C)** Automated analysis parameters optimization **(D)** Variable data acquisition and final IREG calculation.

We have developed an ImageJ plugin (FLEYE) containing four macros: 1) a menu macro (Figure 6A) which controls the other three macros, 2) a macro to create the ROIs of the areas to be analyzed (Figure 6B), 3) a macro to optimize parameters used for the automated analysis (Figure 6C), and 4) the macro that analyzes the images and calculates a regularity index IREG for each eye and a mean IREG per group (Figure 6D). This last macro requires an initial user intervention to select the folders containing the images and ROIs, the folders where final data and graphs will be saved, and to define groups and initial parameters. The macro requires ROIs files with the same name of its corresponding image file to work properly; thus, it is recommended to use the provided “Fleye_ROISv1.2” to create such files easily. The definition of the different parameters used for background subtraction, increase local contrast (surface-like filter) and maxima detection is critical to obtain an adequate classification. FLEYE is ready to work with images with a different resolution from that used here to derive the statistical model. For such purpose, the interface asks for the image pixel length (in microns), and incorporates this value to calibrate the data used in the statistical model. It is recommended to use images with a resolution ≥1.85 μm/pixel (resolution of our images). Furthermore, depending on image features (mainly illumination and resolution) it could be useful to adjust the tolerance and rolling ball radius used for maxima detection. Therefore, we have included a “training macro” (Figure 6B) that helps to define these parameters for automatic counting. This macro requires that the user counts the ommatidia in a restricted area of the eye (Figure 2B), and then an algorithm changes the tolerance and rolling ball radius till a number of maxima similar to that obtained by the user is reached. The user needs to be aware that this macro simply tries to find the parameters considering the area selected, being highly dependent on the adequacy of this area as well as on the user-based quantification. It is recommended to perform this optimization of parameters using images from non-degenerated WT eyes. Multiple images can be used to optimize parameters (one parameter table will be created for each image). If more than one parameter table is loaded for image analysis, the initial parameter dialog-window will be fed with the mean values obtained from the selected tables (these values can be changed before initializing the automated analysis). The final output of FLEYE consists of 7 files with data per image and mean data per group. It also generates graphs plotting IREG for each group and a histogram per group representing the frequency distribution of IREGs.

### Sample validation and model testing

**Figure 7:**
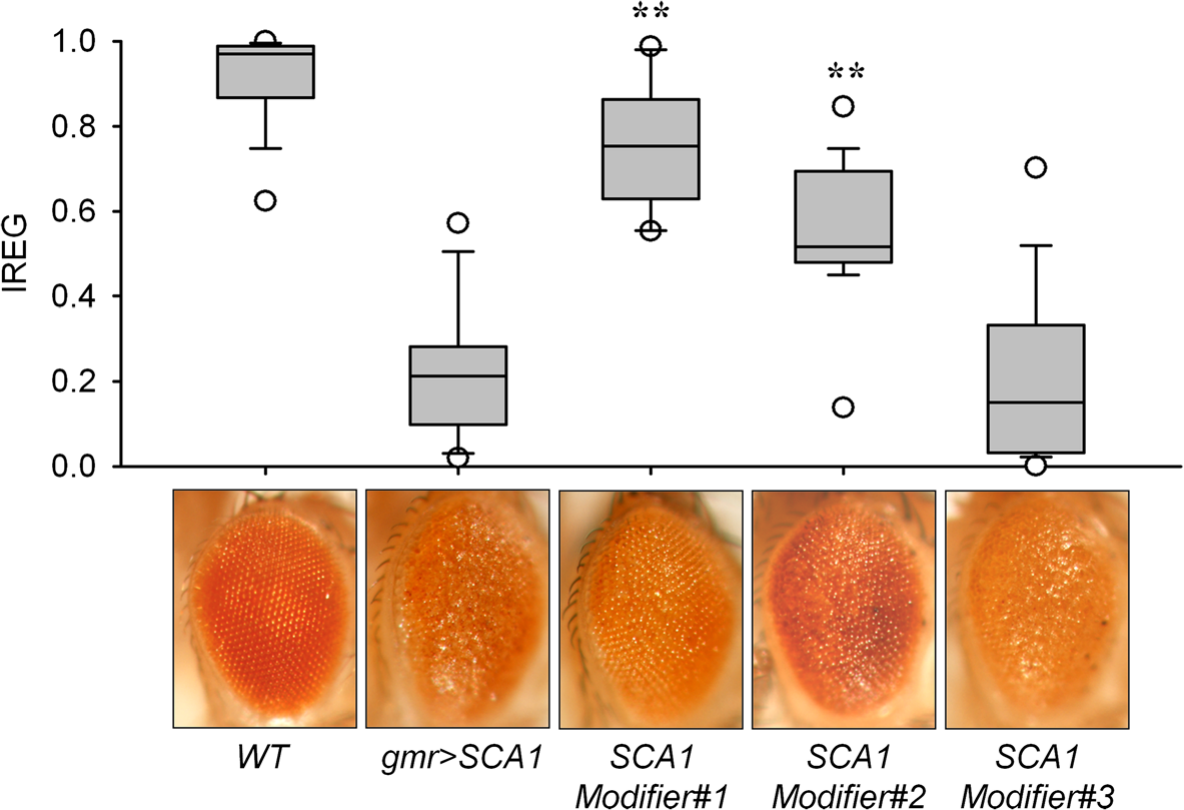
**FLEYE method validation.** IREG value distribution in a sample of fly eyes (n = 85, ≥ 15/genotype). Healthy (*WT*) and degenerated (*gmr > SCA1*) eyes are shown alongside three SCA1 Modifiers. Representative pictures of the eye surface for every genotype are displayed below each box. Statistical significant differences (**) were found between degenerated gmr > SCA1 eyes and the Modifiers #1 and #2, indicating significant phenotypic recovery of the regularity of their eye surface pattern. Statistical significance between experimental classes was assessed by Kruskal-Wallis test and Dunn’s post-hoc tests with p <0.01.

As a proof of concept, we calculated the computed IREG values for five different experimental groups of flies (Figure 7). Alongside the healthy WT and degenerated gmr > SCA1 eyes, three groups showing partially degenerated eyes were included, as an example of various candidate SCA1 modifier genes out of which we find two that rescue and one that does not.

This set of experiments helped us to calculate a percent recovery for a statistically reasonable sample of flies of a given genotype (n ≥ 30 eyes/genotype). As an example, *gmr > SCA1 Modifier#1* showed a 68% recovery as judged by comparing its median IREG with the WT and gmr > SCA1 IREGs. The IREG-based percent recovery slightly overestimates the recovery of the same modifier calculated from retinal thickness measurements (see Figures 1 and 7). This difference is expected since degeneration of lens and of the general architecture of ommatidial surface proceeds at a slower pace, in comparison with the fast disruption of retinal thickness due to massive photoreceptor cell death in this model of *SCA1*. These differences are in a tolerable range, and we are confident that the IREG values estimated by FLEYE and the derived percent recovery are valuable parameters to quickly assess the effect of genetic and pharmacological modifiers of a degenerative condition in the Drosophila eye model. Our method can also be used to appraise treatment variations in a genetically homogeneous fly sample as well as to estimate differences in genetic penetrance of a given genotype. In cases where full degeneration is achieved by the experimental process, resulting in eyes that yield few or no light reflections, FLEYE will assign “complete degeneration” values.

While performing the experiment to validate our method, we have estimated the total time that takes a researcher to analyze an experimental group of 30 flies with our FLEYE plugin: 3 hours from anesthetizing the flies until the IREG plot was statistically assessed and incorporated into a manuscript figure. This time contrasts to the several days needed to analyze retinal parameters from fixed tissues and pictures from SEM, TEM or standard histological sections [8-10]. Another advantage of our methodology is the number of ommatidia explored in each ROI of a single fly (over 200/sample), in comparison with the 30–40 ommatidia counted in each eye when using the deep pseudopupil technique [7].

In contrast to the complex and non-automated methodologies mentioned above that evaluate retinal patterning, a recent work has implemented an automated quantification of the structural features of eye surface obtained from SEM pictures of fly heads using edge detection and boundary-walking algorithms [10]. This method allows for proper statistical analysis of phenotypic differences in fly eye surface, attaining a proper quantitative assessment of retinal distortions. Furthermore, the software using the method algorithm is also freely available. As an advantage, our FLEYE approach is based on the unbiased development of a statistical model that selects the most robust variables accounting for the differences observed between sets of degenerate and wild-type retinas. Moreover, Caudron et al. methodology uses SEM pictures, which imply a lengthy histological procedure and the need of a scanning electron microscope.

Finally, although in the current FLEYE protocol we rely on a simple but manual fly immobilization and image acquisition method, as well as a user-based ROI selection, we can envision simple automatic setups to take well focused and reproducible eye surface pictures of anesthetized flies combined with an easily implemented automatic ROI selection in the near future. Also our method does not require decapitation of flies and can be used to follow the progression of single flies throughout life provided that the immobilization method is reversible. These developments make our method a good candidate for full automation and therefore for potent high throughput screenings in search of therapeutic agents for neurodegenerative diseases.

## Conclusions

In this work we present a novel, easy, and fast method to quantitatively rate the degeneration level of the compound eye of fruit flies with a high degree of reliability and robustness. This new method is based on the acquisition of images from the surface of the eye, the use of automated image analysis tools and a classification algorithm sustained on a built-in statistical model.

The easy-to-use properties of our method, plus the potential to be fully automated, make it a valuable tool for unbiased quantitative estimations of degeneration degrees in genetic or pharmacological screenings using the Drosophila retina as a model system.

In addition, the FLEYE plugin, following the adjustment of model parameters and grid size, could easily be adapted to evaluate the pattern regularity (and their experimental or pathological disruption) of different biological or non-biological origins: from images of crystal structures or beehives, to those of mammalian retina with patterns of cone photoreceptors, or neurons in histological sections of stereotypically organized brain structures.

## Additional files

Additional file 1:
**FLEYE folder containing FLEYE plugin and FLEYE GNU General Public License.**
Additional file 2:
**FLEYE quick guide.**
Additional file 3:
**FLEYE extended user manual.**
