# Supplementary material for: An automated image analysis method to measure regularity in biological patterns: a case study in a Drosophila neurodegenerative model
Source: Mol Neurodegener. 2015 Mar 12;10:9. doi: 10.1186/s13024-015-0005-z (PMC4367968; doi:10.1186/s13024-015-0005-z)
Supplement: Additional file 2: — FLEYE quick guide. [file 13024_2015_5_MOESM2_ESM.pdf]

FLEYE Quick GUIDE

FLEYE is a package of ImageJ macros developed to classify fly eyes attending to the distribution of their ommatidia using images of the eye surface as starting data.
Copyright (C) 2014 Cristina Rueda Sabater, Sergio Díez Hermano, Diego Sánchez Romero, María Dolores Ganfornina Álvarez and Jorge Valero Gómez-Lobo (jorge.valero@cnc.uc.pt).

FLEYE Installation

Unzip the FLEYE.zip file into the plugins folder of FIJI. To solve the issue of malfunction due to FIJI updates we have also included the version of FIJI used when developing FLEYE plugin in FIJI_with_FLEYE.zip files (available for Win32 and Win64; for MacOs we provide a FIJI version that does not include the FLEYE plugin).

Generate folders

It is recommended to create the following folders:

- Original images folder: divide images from the same experimental groups in subfolders.
- Processed images folder: images will be moved to this folder after ROI design. We suggest allocating images from the same experimental groups in separate subfolders.
- Non-processed images folder: images that will not be used for the analysis will be relocated into this folder.
- ROIs folder: ROIs will be stored in this folder.
- Optimized parameters folder: tables containing optimized parameters will be stored here.
- Results folder: Excel result tables and graphs will be stored here.

ROIs design

This macro can be ran through the “FLEYE menu v2” or directly by running the macro “FLEYE ROISv1.2”: Plugins→FLEYE→Fleye ROISv1.2.

1- Select folders: several windows will pop up asking for different folders.

2- ROI design: the first image will open and a window will ask to draw a ROI in the image delimiting the in-focus area. **DO NOT add the ROI to the ROI manager (**if no ROI is drawn the macro will ask whether the user wants to skip this image). Click OK and follow the instructions.

Parameter optimization

This macro can be ran through the “FLEYE menu v2” or directly by running the macro “FLEYE optimizer v4.2”: Plugins→FLEYE→Fleye optimizer v4.2.

1- Specify the image scale: a window will ask for the distance (in microns) of a pixel in your images.

2- Select a representative image: We recommend choosing images of the control conditions.

3- Select a folder to save the table with the optimized parameters for this image.

4- Choose initial parameters. We suggest maintaining default parameters.

5- Place the counting ROI in an adequate position to count. It is important to select a region that represents the images in-focus area.

6- Mark the bright points included in your ROI by pressing the left button of the mouse.

7- Press the right button of the mouse to finish the quantification.

It is highly recommendable to perform this process with at least three representative images to obtain more accurate results.

Analysis

This macro can be ran through the “FLEYE menu v2” selecting “Analysis” or directly by running the macro “Fleye v10.4”: Plugins→FLEYE→Fleye v10.4.

1- Specify the image scale: a window will ask for the distance (in microns) of a pixel in your images.

2- Choose initial options:
- Select “PRE-OPTIMIZED PARAMETERS” if you want to use the optimized parameters obtained above (these parameters are assumed to be already calibrated). Otherwise, you will be able to define the parameters manually or use default ones.
- Select the number of experimental groups to be analyzed.
- Select the number of intervals for frequency histograms.

3- Name the different experimental groups.

4- Select folders.

5- Select optimized parameter files if you previously checked the “PRE-OPTIMIZED PARAMETERS” option in step 2.

6- Let the macro work and go for a walk to a nearby park.

7.- Enjoy the results, write the paper and do not forget to cite our work ;)

GNU General Public License

FLEYE is free software: you can redistribute it and/or modify it under the terms of the GNU General Public License as published by the Free Software Foundation, either version 3 of the License, or (at your option) any later version.

FLEYE is distributed in the hope that it will be useful, but WITHOUT ANY WARRANTY; without even the implied warranty of MERCHANTABILITY or FITNESS FOR A PARTICULAR PURPOSE. See the GNU General Public License for more details.
