# Supplementary material for: An automated image analysis method to measure regularity in biological patterns: a case study in a Drosophila neurodegenerative model
Source: Mol Neurodegener. 2015 Mar 12;10:9. doi: 10.1186/s13024-015-0005-z (PMC4367968; doi:10.1186/s13024-015-0005-z)
Supplement: Additional file 3: — FLEYE extended user manual. [file 13024_2015_5_MOESM3_ESM.pdf]

FLEYE USER GUIDE

FLEYE is a package of ImageJ macros developed to classify fly eyes attending to the distribution of their ommatidia, using images of the eye surface as starting data.
Copyright (C) 2014 Cristina Rueda Sabater, Sergio Díez Hermano, Diego Sánchez Romero, María Dolores Ganfornina Álvarez and Jorge Valero Gómez-Lobo (jorge.valero@cnc.uc.pt).

FLEYE Installation

The package includes three ImageJ macros that will allow the user to select in each image the regions of interest (ROI) for analysis, optimize parameters and analyze the images. To install the package unzip the FLEYE.zip file into the plugins folder of FIJI. To solve the issue of malfunction due to FIJI updates we have also included the version of FIJI used when developing FLEYE plugin in FIJI_with_FLEYE.zip files (available for Win32 and Win64; for MacOs we provide a FIJI version that does not include the FLEYE plugin).

Preparing images for analysis

Before playing around with the macro it is recommended to create the following folders:

- Original images folder: this folder should contain the images to be analyzed. It is recommendable to create subfolders where images from similar experimental groups are stored.
- Processed images folder: images will be moved to this folder after ROI design. This is the way this macro has to allow continuing with the work after interruptions. We suggest creating subfolders to allocate images from similar experimental groups.
- Non-processed images folder: images that will not be used for the analysis will be relocated into this folder.
- ROIs folder: ROIs will be stored in this folder.
- Optimized parameters folder: tables containing optimized parameters will be stored here if optimization is performed.
- Results folder: Excel result tables and graphs will be stored here. Subfolders with the name of each experimental group will be automatically created to store corresponding tables and graphs. An extra subfolder named “Mean” will be generated where mean data from the different experimental groups and graphs will be located.

Running the FLEYE macro:

To run the macro start FIJI and click on Plugins→FLEYE→FLEYE menu v2. An initial window will appear showing information about the license of the package. After clicking in the "OK" button the initial menu of FLEYE will open offering 4 different options:

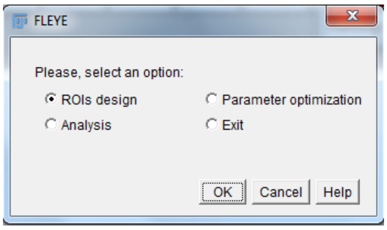

- ROIs design
- Parameter optimization
- Analysis
- Exit

Select the adequate option and then click on "OK" button, a confirmation window will appear (click on Yes to continue).

ROIs design

This macro can be run through the “FLEYE menu v2” or directly by running the macro “Fleye ROISv1.2”: Plugins→FLEYE→Fleye ROISv1.2.

A window disclaiming the FLEYE GNU license will appear. After clicking on "OK" several windows will pop up sequentially asking for different folders:
1.- The folder containing the images (this folder should include just images). We suggest the use of different folders for each experimental group.
2.- A folder where processed images will be automatically placed. It is highly recommendable to use one folder per experimental group.
3.- A folder to store the images that will not be used.
4.- A folder where newly generated ROIs will be saved.
After selecting the different folders, the first image will open. This macro uses the “Bio-Formats Importer” to open images, therefore this plugin is required for normal functioning. “Bio-Formats Importer” supports different image formats (please check the list of file formats supported here: http://www.openmicroscopy.org/site/support/bio-formats5/supported-formats.html).

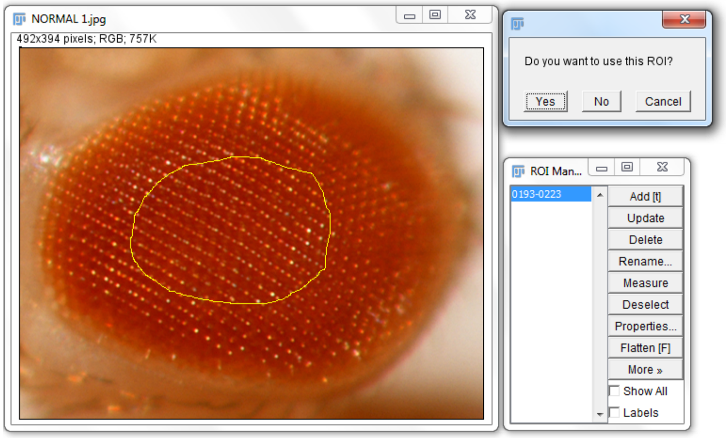

The polygon tool will be automatically selected and a window will ask the user to draw a ROI in the image. Other tools for ROI design can be selected if desired. **DO NOT add the ROI to the ROI manager;** the macro will do it automatically after clicking on “OK”, showing the ROI in the image and asking whether the user wants to use the ROI. By clicking on “Yes” the ROI will be saved to the ROIs folder as a zip file with the same name than the image. By clicking on “NO” the macro will erase the ROI and allow the user to draw a new one by repeating the process. If no ROI is drawn the macro will ask whether the user wants to skip this image. If the answer is “Yes” the image will be moved to the non-processed image folder and no ROI will be stored at the ROIs folder (this image will not be used for further analysis). Finally, a window asking whether the user wants to continue will appear. By clicking in “NO” the macro will finish. As the processed images are moved from one folder to another, this macro allows interruptions and continuing the ROI design without repeating the process in previously processed images if the folder is adequately selected at the beginning.

Parameter optimization
This macro can be run through the “FLEYE menu v2” or directly by running the macro “Fleye optimizer v4.2”: Plugins→FLEYE→Fleye optimizer v4.2.

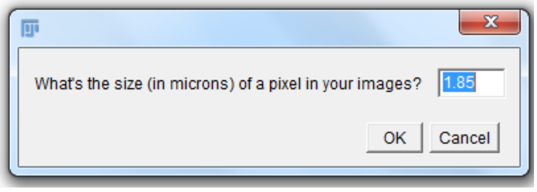

A window disclaiming the FLEYE GNU license will appear. After clicking on "OK" a window will ask for the distance (in micrometers) of a pixel in the images that are going to be use. The value by default is 1.85, which is the value of the images used for the development of the FLEYE plugin. It is very important to calibrate the images, as the macro needs to normalize the pixel scale of the images to classify the images adequately. After clicking on “OK”, a window asking to select a representative image will appear. We recommend choosing images of the control conditions. Once the image is selected a new window will appear to allow selecting a folder where the table with the optimized parameters for this image will be saved. Then, a window with the initial parameters will be shown (we suggest maintaining the default parameters):

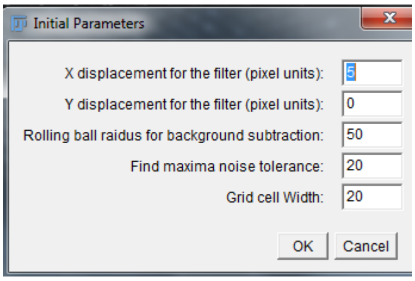

- X displacement for the filter: indicates the number of pixels that an intensity-inverted copy of the image will be displaced horizontally (x axis) for the surface filter.
- Y displacement for the filter: indicates the number of pixels that an intensity-inverted copy of the image will be displaced horizontally (y axis) for the surface filter.
- Rolling ball radius for background subtraction: this is a parameter related to the "Background subtraction" plugin (for further information check: http://imagejdocu.tudor.lu/doku.php?id=gui:process:subtract_background)
- Find maxima noise tolerance: this is a parameter related to the way in which local maxima will be selected when using the "Find Maxima" plugin (for further information check: http://imagej.nih.gov/ij/docs/menus/process.html#find-maxima).
- Grid cell Width: it is the width of the cells of the grid used to analyze the distribution of local maxima. We do not recommend changing this parameter, as it is highly important for the model. We decided to include the possibility of changing the Grid cell Width because it could be useful for the analysis of other regular structures (in such a case the parameters of the statistical model should be determined again to allow for an adequate classification).

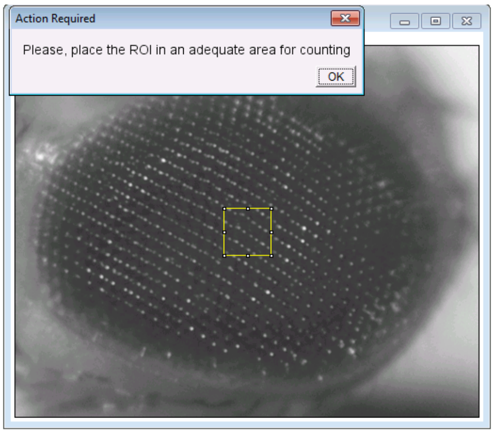

A new window will appear asking to place a squared ROI in an adequate region to count. This ROI can be resized and it is also possible to create a new ROI with a different shape. It is important at this point to select a region that could be representative of the image and its variability (containing different grades of brightness).

After clicking on "OK" you will be able to mark the elements of interest included in your ROI by pressing the left button of the mouse. Points marked outside of the ROI will be also counted, so please be careful and avoid such type of error. Points are added to the ROI Manager so you will be able to erase points by selecting them in the ROI Manager and using the Delete option. Once you have marked all the elements you will have to press the right button of the mouse to finish the selection. Then, a confirmation window will appear and after clicking on "Yes" the macro will start to search for the parameters that produce the same or more similar number of counts to those obtained by the user. In the Log window the counts obtained by the program and the user can be checked while processing. After finishing, the macro will allow you to check the parameters table. Finally, the table will be saved as a text file (.txt) to the parameters folder.

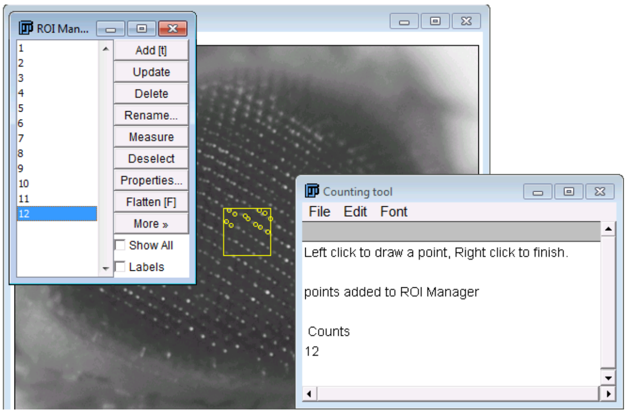

It is highly recommendable to perform this process with at least three representative images to obtain more accurate results.

Analysis

This macro can be run through the "FLEYE menu v2" selecting "Analysis" or directly by running the macro "Fleye v10.4": Plugins→FLEYE→Fleye v10.4.

A window disclaiming the FLEYE GNU license will appear. After clicking on "OK" a window will ask for the distance (in micrometers) of a pixel in the images (see above). After clicking on “OK”, a window with several options will appear:

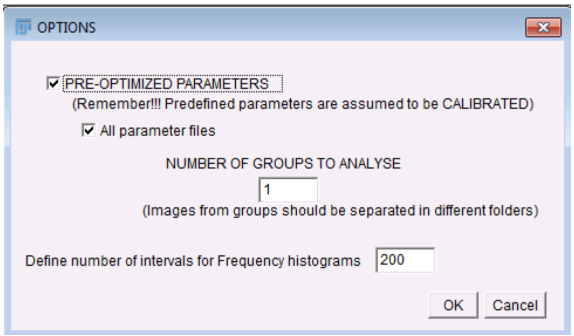

- Select “PRE-OPTIMIZED PARAMETERS” if you want to use the parameters obtained using the parameters optimization option included in FLEYE. Be aware that these parameters are assumed to be already calibrated. If this option is selected, you will be able to either use all the parameter files contained in the parameters folder or select just some of them.

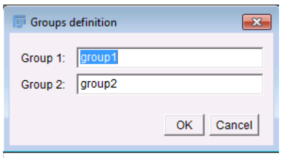

- Select also the number of experimental groups to be analyzed. Remember that the images from different groups should be located in different folders.
- Finally, you will have to select the number of intervals (bars) that will be used to divide the “regularity index” axis when creating the frequency histograms (see bellow).

After selecting the options a new window will give you the opportunity of naming the different experimental groups. Then, the folders containing the images of each group and the ROIs, the folder where results will be stored, and the folder containing the parameter files (if this option has been previously selected) must be selected. A window asking to select the parameter files to be used will appear. Check the name of the files you want to use (the mean value of the parameters from the different files will be used for the analysis). A parameter window will pop up to allow checking and changing values (see above for parameters meaning). Then, the analysis will start and different tables and graphs will appear and be saved to the results folder separated in different subfolders. The subfolders correspond to the different experimental groups, and one folder will contain mean data and graphs from all groups. The group subfolders store three different Excel files (classification, distance to the center of mass and nearest neighbor) and two files containing the graphs (accumulated probability and frequency histogram). The “Means” folder contains just one “Mean_Accumulated probability” graph file, the same Excel files mentioned above (now with mean data per group) and an extra one containing the parameters used for the analysis.

Description of data tables:

Mean tables and graphs show mean values per group.

<u>Nearest Neighbor</u>

- Date: the date in which the analysis was initiated
- Time: the time in which the analysis was initiated
- Image/group: name of the image/group analyzed.
- Points: number of elements identified inside the ROI.
- Nearest Neighbor: mean distance between each single point and the closest point (the unit of this parameter is normalized pixel, if you want to know its value in microns you just have to multiply its value by the factor 1.85).
- StdDv: standard deviation of the mean “Nearest Neighbor” distance.
- Var: variance of the Nearest Neighbor distances.

<u>Distance to the center of mass:</u>

- Date: the date in which the analysis was initiated
- Time: the time in which the analysis was initiated.

- Image/group: name of the image/group analyzed.
- Cells: number of cells containing points.
- Mean: mean value of the distances between the center of each particular cell and its center of mass (in normalized pixels, if you want to know its value in microns you have to multiply its value by the factor 1.85).
- StdDv: standard deviation of the mean distance to the center of mass.
- Var: variance of the distances to the center of mass.
- Skew: skewness of the distances to the center of mass.

<u>Classification:</u>

- Date: the date in which the analysis was initiated.
- Time: the time in which the analysis was initiated.
- Image/group: name of the image/group analyzed.
- DEGCALL (degeneration call): this is a parameter used to classify images into to categories: degenerated (values close to 1) and non-degenerated (values close to 0).
- IREG (index of regularity): this is a parameter that indicates the regularity of the distribution of the elements inside the system. It ranges between 1 for the most regular distribution and 0 for the most irregular one.
- PPs (PP0, PP1, PP2, PP3, PP4): parameters that indicate the probability of a given system to belong to 5 different groups from 0 to 4, being 0 the group corresponding to the most regular one (control or non-degenerated) and the group 4 the most irregular one (showing the greatest degeneration).

group1_Nearest Neighbour

File Edit Font

Date	time	Image	Points	Nearest Neighbour	StdDv	Var
9/26/2014	10:34:24	NORMAL 1.jpg	251	8.4106	2.7898	7.7522
9/26/2014	10:34:24	NORMAL 2.jpg	139	10.2882	3.022	9.0665
9/26/2014	10:34:24	NORMAL 3.jpg	171	9.4187	2.6191	6.8197

group1_Distance to the center of mass

File Edit Font

Date	time	Image	Cells	Mean	StdDv	Var	Skew
9/26/2014	10:34:24	NORMAL 1.jpg	70	3.4028	1.9363	3.7494	0.6884
9/26/2014	10:34:24	NORMAL 2.jpg	45	3.572	2.2064	4.8683	0.7787
9/26/2014	10:34:24	NORMAL 3.jpg	49	3.4281	1.7742	3.1479	1.2932

group1_Classification

File Edit Font

Date	time	Image	DEGCALL	IREG	PP0	PP1	PP2	PP3	PP4
9/26/2014	10:34:24	NORMAL 1.jpg	1.0001E-8	0.7817	0.1295	0.868	0.002501	3.3340E-13	9.9757E-9
9/26/2014	10:34:24	NORMAL 2.jpg	5.6070E-8	0.7555	0.02985	0.9623	0.007817	3.8380E-13	5.5632E-8
9/26/2014	10:34:24	NORMAL 3.jpg	4.8552E-14	0.983	0.9319	0.06814	1.7115E-6	3.6092E-23	4.8552E-14

Graph descriptions:

<u>Accumulated probability:</u>

These graphs represent the accumulated probability for each eye to belong to 5 different groups from 0 to 4, being the group 0 the most regular one and the group 4 the most irregular. Different group probabilities are represented by different colors: PP0 in green, PP1 in blue, PP2 in yellow, PP3 in orange and PP4 in red.

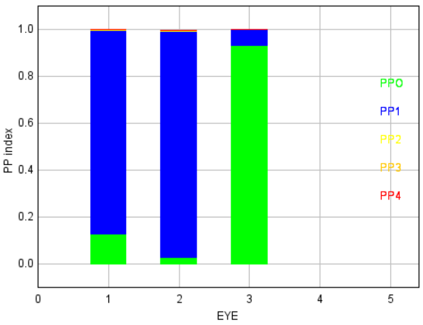

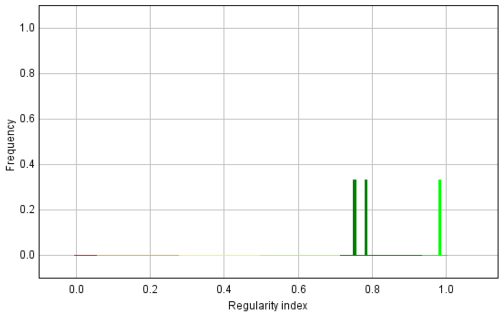

<u>Frequency histogram:</u>
In this graph each bar represents a particular range of the regularity index value, whose size is determined by the number of ranges selected at the beginning of the analysis, 200 by default. Red/orange colors represent low regularity ranks, yellow colors intermediate ranks and blue/green colors the high ranks.

<u>GNU General Public License</u>
FLEYE is free software: you can redistribute it and/or modify it under the terms of the GNU General Public License as published by the Free Software Foundation, either version 3 of the License, or (at your option) any later version.

FLEYE is distributed in the hope that it will be useful, but WITHOUT ANY WARRANTY; without even the implied warranty of MERCHANTABILITY or FITNESS FOR A PARTICULAR PURPOSE. See the GNU General Public License for more details.
